# Long-term Elevation of Complement Factors in Cerebrospinal Fluid of Patients With Borna Disease Virus 1 Encephalitis

**DOI:** 10.1093/infdis/jiae183

**Published:** 2024-04-09

**Authors:** Markus Bauswein, Saida Zoubaa, Martina Toelge, Lisa Eidenschink, Markus J Riemenschneider, Bernhard Neumann, De-Hyung Lee, Ehab Eid, Dennis Tappe, Hans Helmut Niller, André Gessner, Barbara Schmidt, Sigrid Bülow, Klemens Angstwurm

**Affiliations:** Institute of Clinical Microbiology and Hygiene, University Hospital Regensburg, Regensburg, Germany; Department of Neuropathology, University Hospital Regensburg, Regensburg, Germany; Institute of Clinical Microbiology and Hygiene, University Hospital Regensburg, Regensburg, Germany; Institute of Clinical Microbiology and Hygiene, University Hospital Regensburg, Regensburg, Germany; Department of Neuropathology, University Hospital Regensburg, Regensburg, Germany; Department of Neurology, Donau-Isar-Klinikum Deggendorf, Deggendorf, Germany; Department of Neurology, University of Regensburg, Bezirksklinikum, Regensburg, Germany; Department of Neurology, University of Regensburg, Bezirksklinikum, Regensburg, Germany; Department of Neurology, University of Regensburg, Bezirksklinikum, Regensburg, Germany; Bernhard Nocht Institute for Tropical Medicine, Hamburg, Germany; Institute of Medical Microbiology and Hygiene, University of Regensburg, Regensburg, Germany; Institute of Clinical Microbiology and Hygiene, University Hospital Regensburg, Regensburg, Germany; Institute of Medical Microbiology and Hygiene, University of Regensburg, Regensburg, Germany; Institute of Clinical Microbiology and Hygiene, University Hospital Regensburg, Regensburg, Germany; Institute of Medical Microbiology and Hygiene, University of Regensburg, Regensburg, Germany; Institute of Clinical Microbiology and Hygiene, University Hospital Regensburg, Regensburg, Germany; Department of Neurology, University of Regensburg, Bezirksklinikum, Regensburg, Germany

**Keywords:** BoDV-1, zoonosis, complement system, immunopathogenesis, biomarkers

## Abstract

**Background:**

Borna disease virus 1 (BoDV-1) causes rare but severe zoonotic infections in humans, presenting as encephalitis. The case-fatality risk is very high and no effective countermeasures have been established so far. An immunopathology is presumed, while data on immune responses in humans are limited. Evidence of a role of the complement system in various neurological disorders and in viral infections of the central nervous system is increasing and specific inhibitors are available as therapeutic options.

**Methods:**

In this study, we investigated factors of the complement system in the cerebrospinal fluid (CSF) of patients with BoDV-1 infections (n = 17) in comparison to noninflammatory control CSF samples (n = 11), using a bead-based multiplex assay. In addition, immunohistochemistry was performed using postmortem brain tissue samples.

**Results:**

We found an intrathecal elevation of complement factors of all complement pathways and an active cascade during human BoDV-1 infections. The increase of certain complement factors such as C1q was persistent, and C3 complement deposits were detected in postmortem brain sections. Intrathecal complement levels were negatively correlated with survival.

**Conclusions:**

Further investigations are warranted to clarify whether targeting the complement cascade by specific inhibitors might be beneficial for patients suffering from severe BoDV-1 encephalitis.

## Teaser

Complement factors are persistently elevated in cerebrospinal fluid of patients with Borna disease virus 1 encephalitis and are deposited in brain tissue. The activation of the complement system might impact the disease course and warrants further investigation.

Borna disease virus 1 (BoDV-1) is a highly neurotropic enveloped RNA virus with a linear, nonsegmented, single-stranded, negative-sense genome of approximately 8.9 kilobases (order *Mononegavirales*, family *Bornaviridae*, genus *Orthobornavirus*, species *Orthobornavirus bornaense*) that is well-known for causing severe spillover infections in mammals [1]. The bicolored white-toothed shrew (*Crocidura leucodon*) serves as natural reservoir [2]. In addition to the long-known dead-end animal hosts such as horses and sheep, in 2018 humans also were proven to be susceptible to BoDV-1 infections, which lead to severe encephalitis [3, 4]. In the known endemic regions in southern and eastern Germany, approximately 50 new or retrospective cases of human infections have been reported since 2018 [5–9]. The case-fatality risk is very high and no effective countermeasures have been established so far, despite off-label treatment attempts with antivirals (favipiravir, ribavirin) and additional immunosuppressive medication [10]. BoDV-1 does not cause a cytopathic effect in cell culture and establishes a persistent infection in the reservoir and certain (experimentally) infected hosts [11]. Evidence from animal experiments suggests an immunopathogenesis in symptomatic spillover hosts that has been primarily attributed to T-cell responses. For example, the infection of mice lacking CD8 T cells was not associated with signs and symptoms of the disease [12]. Furthermore, prophylactic or early administration of different immunosuppressive compounds prevented neurological disease in animals [13–15]. In human patients, there are limited observations that at least indicate a strong immune activation during the disease: Brain tissue sections show parenchymal and perivascular infiltrates of CD4 and CD8 lymphocytes [16]. Patients with immunosuppression due to another medical condition tended to survive longer than patients without immunosuppression [6]. A long-term survivor of a symptomatic BoDV-1 infection received cyclosporine A as immunosuppressive medication after a liver transplantation [4]. A recent study found increased proinflammatory cytokines such as interferon-γ, interleukin (IL-) 6, and IL-12p40 as well as elevated chemokines (CCL-2, CCL-5, CXCL-10, IL-8) in both serum and cerebrospinal fluid (CSF) samples of patients with BoDV-1 infection [17].

Data on the role of the complement system during BoDV-1 infections, however, are lacking. The complement system is a network of proteins in plasma and on cell surfaces that was first described to “complement” antibodies in killing bacteria [18]. Besides host defense against infections, it contributes to tissue homeostasis, the pruning/elimination of synapses within the central nervous system (CNS), and is involved in the polarization of proinflammatory astrocytes [19, 20]. In addition to the liver, certain cells within the CNS such as astrocytes and microglia have been shown to be a source of complement factors during various neurological diseases [21]. There is growing evidence for a role of the complement system in neurological diseases such as neuromyelitis optica spectrum disorder (NMOSD), myasthenia gravis, autoimmune encephalitis, and neurodegenerative disorders [22].

In the present study, our aim was to measure complement factors in CSF samples of patients with BoDV-1 encephalitis and to evaluate their potential as additional diagnostic and prognostic markers or as possible targets for treatment.

## METHODS

### Patients and Samples

Thirty-five diagnostic leftover CSF samples of 17 individual patients with confirmed BoDV-1 infection were included in the analysis (Supplementary Table 1). Twenty-eight samples of 10 individual patients were available at the Institute of Clinical Microbiology and Hygiene of the University Hospital Regensburg, Germany (center 1); 7 samples of 7 additional patients were provided by the Bernhard Nocht Institute for Tropical Medicine, Hamburg, Germany (center 2). Samples were collected between 1999 and 2022. More information is available in the Supplementary Materials.

As controls, 11 diagnostic leftover CSF samples of 11 individual patients who underwent diagnostic lumbar puncture in the years from 2020 to 2021 for the exclusion of an infection of the CNS were used. More information is available in the Supplementary Materials.

For comparison with tick-borne encephalitis (TBE), 7 diagnostic leftover samples of patients with confirmed TBE from the years 2014 to 2020 were used. TBE was diagnosed by a positive TBE-virus-specific immunoglobulin G CSF serum antibody index and a positive CSF immunoglobulin M.

CSF samples were stored at −20 °C prior to testing. Samples provided by center 2 had to be shipped (on dry ice) before measurement.

The analysis of samples for BoDV-1 infection was approved by the ethical commission of the Faculty for Medicine, University of Regensburg, Germany (reference number 18-1248-101). The retrospective analysis of pseudonymized diagnostic leftover samples for the analysis of immunological biomarkers was approved by the ethical commission of the Faculty for Medicine, University of Regensburg, Germany (reference number 18-1269-101).

### Bead-Based Multiplex Assay for Complement Factors

Complement factors in CSF samples were measured using the MILLIPLEX Human Complement Magnetic Bead Panel 1 (Merck, Darmstadt, Germany; #HCMP1MAG-19K-07: C2, C4b, C5, C5a, CFD, CFI, MBL), the MILLIPLEX Human Complement Magnetic Bead Panel 2 (Merck; #HCMP2MAG-19K-06: C1q, C3, C3b_iC3b, C4, CFB, CFH), and the MILLIPLEX Human Complement Magnetic Bead Panel 2 (Merck; #HCMP2MAG-19K-01: C3) according to the manufacturer’s instructions. A detailed description of the method is available in the Supplementary Materials. Minimum detectable concentrations as they were calculated by the manufacturer are as follows: C2: 0.25 ng/mL; C4b: 0.28 ng/mL; C5: 0.68 ng/mL; C5a: 0.0023 ng/mL; CFD: 0.016 ng/mL; CFI: 0.15 ng/mL; MBL: 0.036 ng/mL; C1q: 0.033 ng/mL; C3: 0.080 ng/mL; C3b/iC3b: 2.385 ng/mL; C4: 0.132 ng/mL; CFB: 0.020 ng/mL; CFH: 0.115 ng/mL. Samples with values above the upper limit of quantification were diluted to receive a result within the limit of quantification.

### Immunohistochemistry

Brain tissue samples from autopsies were available for 3 patients with fatal BoDV-1 encephalitis (patients 7, 8, and 10 of this manuscript). Brain tissue samples from the routine autopsy of a patient, who died from cardiac arrest and showed no central nervous inflammation/infection, served as negative control. Tissue samples were fixed in 4% buffered formalin, embedded in paraffin, and cut into 2-µm-thick sections. Immunostainings were performed using a fully automated staining system (Ventana BenchMark, Roche, Basel, Switzerland). The standardized protocol included epitope retrieval for 24–48 minutes at 110 °C in citrate (pH 6.0). Endogen peroxidase activity was blocked by 1× Dako Peroxidase-Blocking Solution (Dako, Jena, Germany; #S2023) for 10 minutes. The sections were then incubated with a rabbit monoclonal antibody directed against human complement C3 (Invitrogen, Thermo Fisher Scientific, Waltham, Massachusetts, USA; #MA5-38352; clone 4D12; 1:2000 dilution). The monoclonal mouse antibody Bo18 directed against the BoDV-1 nucleoprotein [23] (generously provided by Sibylle Herzog, University of Giessen, Germany) was used to stain virus-infected cells as described previously (1:500 dilution in 0.05 M Tris-buffered saline, pH 7.6) [24]. The antigen-antibody complexes were detected with the OptiView DAB IHC Detection Kit (Roche; #06396500001 Ventana, Roche; #760-700). Slides were counterstained with hematoxylin and bluing reagent. Double immunofluorescence was performed in order to examine co-localization of C3 with CD163, glial fibrillary acidic protein (GFAP), and the neuronal nuclear antigenNeuN, respectively. The following antibodies were used: anti-CD163 (Novocastra, Newcastle upon Tyne, UK; #NCL-CD163; 1:1000 dilution), anti-GFAP (Diagnostic BioSystems, Pleasanton, California, USA; #Mob064; clone GA5; 1:200 dilution), anti-NeuN (Cell Signaling, Danvers, Massachusetts, USA; #24307; clone D4G40; 1:50 dilution). C3 was detected using the fluorochrome dye Opal 520 (AKOYA Bioscience, Marlborough, Massachusetts, USA; #FP1487001KT), while CD163, GFAP, and NeuN were detected using Opal 650 (AKOYA Bioscience; #FP1496001KT).

### Statistical Analysis

Data were analyzed and figures were created using GraphPad Prism version 10.1.0 (GraphPad Software, San Diego, California, USA) and R version 4.3.1 (R Foundation for Statistical Computing, Vienna, Austria). More information is available in the Supplementary Materials.

## RESULTS

### Characteristics of Patients and Samples

For the retrospective analysis of complement factors in CSF, 35 diagnostic leftover CSF samples of 17 individual patients with confirmed BoDV-1 infection were included. Median age of patients was 58 years (interquartile range [IQR]: 37–66 years); 7 of the 17 patients (41%) were female (Supplementary Table 1). Age and sex are not revealed on an individual basis due to ethical reasons.

As controls, 11 diagnostic leftover CSF samples of 11 individual patients who underwent diagnostic lumbar puncture in the years from 2020 to 2021 to rule out an inflammatory disease of the CNS were used. Median age of control patients was 51 years (IQR: 39.5–61.5 years); 5 patients were female (45%).

### Complement Factors of All 3 Pathways Are Early Elevated in the CSF of Patients With BoDV-1

In a first analysis, complement factors were determined in the first available CSF sample of each patient with BoDV-1 infection and were compared to CSF samples of the control group. The first available CSF samples of patients diagnosed with BoDV-1 encephalitis were acquired on average 13 days after hospitalization (range: 0–35 days).

**Figure 1.**
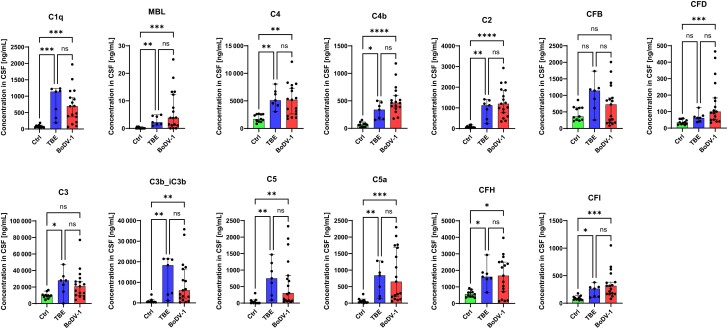
Complement factors are elevated in cerebrospinal fluid (CSF) of patients diagnosed with tick-borne encephalitis (TBE) and Borna disease virus 1 (BoDV-1). CSF samples of patients with TBE (n = 7) and confirmed BoDV-1 infection (n = 17) were examined by a Luminex bead-based multiplex assay and were compared to CSF samples of patients without infections of the central nervous system (Ctrl; n = 11). For patients with BoDV-1 infection and available follow-up CSF samples, only the first available sample was included in the analysis. Depicted are individual values; bars represent the median, and error bars show the 95% confidence interval of the median. For each analyte, a Kruskal-Wallis test followed by Dunn multiple comparisons test was computed. The asterisks indicate statistically significant differences: * *P* ≤ .05; ** *P* ≤ .01; *** *P* ≤ .001; **** *P* ≤ .0001; ns (not significant) *P* > .05.

**Figure 2.**
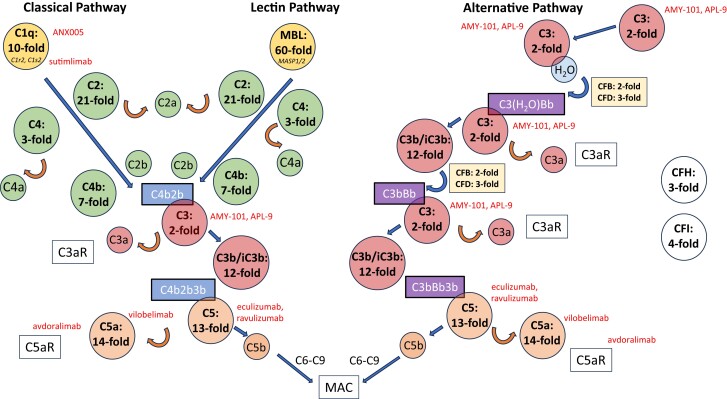
Fold induction of complement factors within the 3 complement pathways in the first available cerebrospinal fluid (CSF) samples of patients with Borna disease virus 1 encephalitis. Fold induction of the median in comparison to control CSF samples is shown with the exception of MBL and C5, for which fold induction of the mean is given for mathematical reasons since the median of control samples equals 0. Potential inhibitors of complement factors are shown in red.

Complement factors C1q and MBL as initiation factors of the classical and the lectin pathway, respectively, were both significantly elevated in CSF during BoDV-1 infection (Figure 1). For C1q, a 10-fold induction of the median (695 ng/mL; 95% confidence interval [CI]: 371–955 ng/mL) was measured, while the MBL concentration was 3.83 ng/mL (median; 95% CI: 1.07–12.25 ng/mL), compared to a median of 0.00 ng/mL (95% CI: .00–.61  ng/mL) for control samples (Figure 2). As consecutive factors of the classical and the lectin pathway, C4 (3-fold induction of median), C4b (7-fold induction of median), and C2 were also significantly elevated, with the 21-fold median induction of C2 to 1200 ng/mL (95% CI: 771–1855 ng/mL) representing the most abundant increase of all complement factors included in the measurement. Among the factors associated with the alternative pathway, CFD was significantly elevated (3-fold induction of median), whereas the increase of CFB did not reach statistical significance. Regarding uncleaved C3, the groups of control patients and patients with BoDV-1 did also not statistically differ, while cleaved C3b_iC3b was significantly elevated in patients diagnosed with BoDV-1 (12-fold induction of median), indicative for an increased activity of C3 convertases during BoDV-1 infection. In line, cleaved factor C5a was in median elevated 14-fold (650 ng/mL; 95% CI: 194–1680 ng/mL), while the concentration of uncleaved factor C5 showed a 10-fold median induction (303 ng/mL; 95% CI: 77–835 ng/mL). Inhibitory factors CFH and CFI were elevated 3- and 4-fold, respectively.

In summary, an early elevation of complement factors of all pathways as well as an increase of cleaved factors indicating active C3 and C5 convertases was observed in the first available CSF samples of patients with BoDV-1 encephalitis.

### No Difference in Elevation of CSF Complement Factors During BoDV-1 Infection and TBE

In a next step, we addressed the question of whether the elevation of complement factors in CSF samples of patients with BoDV-1 infection resulted in a specific biomarker pattern distinguishing BoDV-1 encephalitis from other viral infections of the CNS. For this purpose, the first available CSF samples of patients with BoDV-1 infection were compared to diagnostic CSF samples of 7 patients with confirmed TBE from the years 2014–2020. Median age of patients with TBE was 49 years (IQR: 46–59.6 years), 4 patients were female (57%).

**Figure 3.**
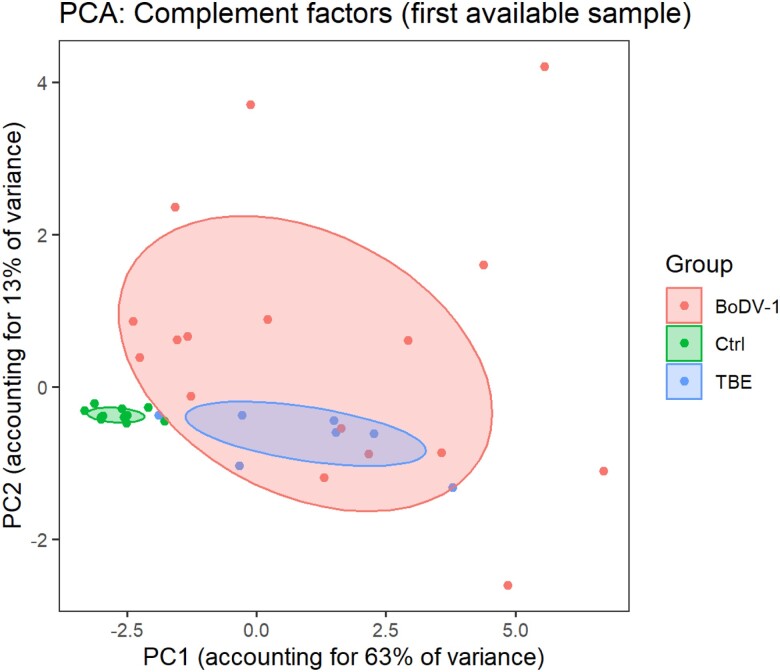
Principal component analysis (PCA) of complement factors shows overlap of patients with tick-borne encephalitis (TBE) and Borna disease virus 1 (BoDV-1) infection, while both groups are distinct from control samples (Ctrl). Based on complement factors measured in the first available cerebrospinal fluid sample of all patients, PCA was performed. Individual data points are shown in projection onto principal component (PC) 1 (accounting for 63% of total variance in the data) and PC2 (accounting for 13% of total variance in the data) as x- and y-axis, respectively. Group-related ellipses were computed as polygons, based at a confidence level of 60%.

A significant group difference between patients with TBE and BoDV-1 was not found for any of the measured complement factors, based on Kruskal-Wallis tests followed by Dunn multiple comparisons tests (Figure 1). Additionally, a principal component analysis was performed (Figure 3). Projecting individual data points onto principal component (PC) 1 (accounting for 63% of total variance in the data) and PC2 (accounting for 13% of total variance in the data) and computing group-related ellipses at a confidence level of 60% revealed an overlap of patients with TBE and BoDV-1. In conclusion, complement factors do not seem to be suitable to differentiate BoDV-1 infections from TBE, but their levels in BoDV-1 infections are clearly distinct from control samples.

### Concentration of Complement Factors in CSF of Patients With BoDV-1 Encephalitis Negatively Correlates With Survival

**Figure 4.**
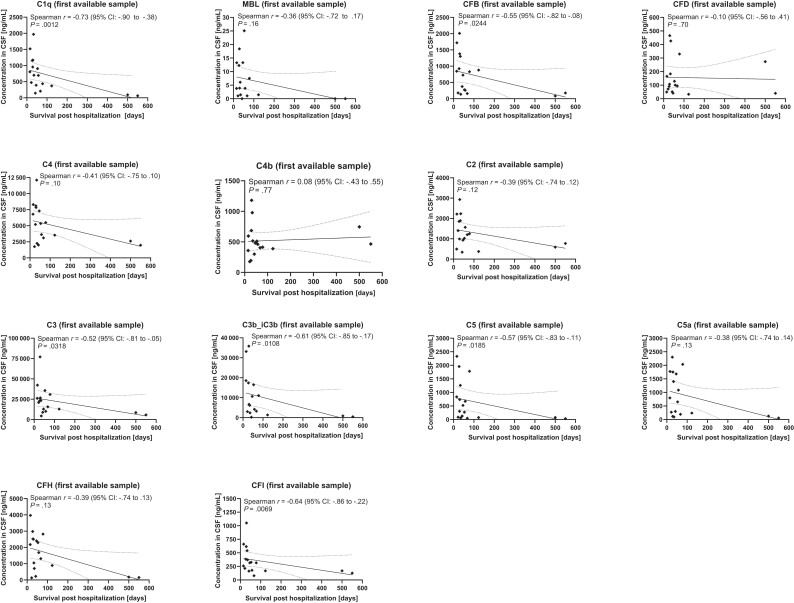
Intrathecal complement levels in Borna disease virus 1 (BoDV-1) encephalitis are negatively correlated with survival. Survival after hospital admission is shown on the x-axis, while concentrations of complement factors in the first available cerebrospinal fluid (CSF) samples (sampling 0–35 days after hospitalization) of patients with BoDV-1 encephalitis are given on the y-axis. Spearman correlation tests and a linear regression (solid line; dotted lines represent 95% confidence band of best-fit line) were performed.

In addition, possible associations of interindividual differences of intrathecal complement factors in patients with BoDV-1 encephalitis with the clinical course of the disease were investigated. As an objective clinical parameter, survival after hospital admission was used. Concentrations of complement factors C1q (Spearman *r* = −0.73; 95% CI: −.90 to −.38; *P* = .0012), CFB (Spearman *r* = −0.55; 95% CI: −.82 to −.08; *P* = .0244), C3 (Spearman *r* = −0.52; 95% CI: −.81 to −.05; *P* = .0318), C3b_iC3b (Spearman *r* = −0.61; 95% CI: −0.85 to −0.17; *P* = .0108), C5 (Spearman *r* = −0.57; 95% CI: −.83 to −.11; *P* = .0185), and CFI (Spearman *r* = −0.64; 95% CI: −.86 to −.22; *P* = .0069) in the first available CSF samples (sampling 0–35 days after hospitalization) were negatively correlated with survival at a statistically significant level (Figure 4*A*). Time of sampling was not significantly correlated with survival, making a potential sampling bias unlikely (Supplementary Figure 1). In addition, the effect was not based on the 2 outliers provided by center 2 with survival ≥500 days alone and it was not based on differences between center 1 and center 2 alone, evident by the negative correlations in the subgroup analysis of samples provided by center 1 only (Supplementary Figure 2). In conclusion, the level of complement factors in early CSF samples seems to be a negative predictor of survival.

### Long-term Elevation of Complement Factors in CSF of Patients With BoDV-1 Encephalitis

**Figure 5.**
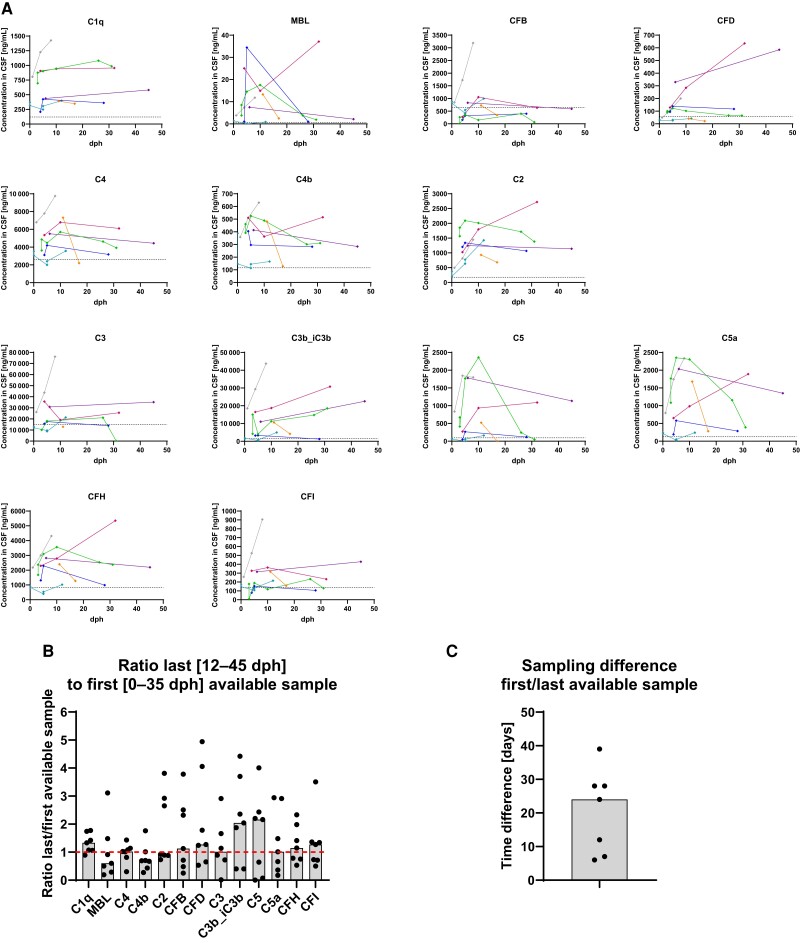
Complement factors remain elevated in patients with Borna disease virus 1 (BoDV-1) infection during the time course of the disease. *A*, Time courses of complement factors in the cerebrospinal fluid (CSF) of 7 patients with confirmed BoDV-1 infection and available follow-up CSF samples (with a sampling difference of >1 day) are shown (dph: days post hospitalization). The results of the individual patients are displayed as dots and lines in the same color. Dotted horizontal lines give the upper 95% confidence limit of the median of the 11 tested negative controls (patients without central nervous infections). *B*, Ratios of complement factors in the last (sampling 12–45 days after hospitalization) and first (sampling 0–35 days after hospitalization) available sample were calculated. The dotted line represents a ratio of 1, bars indicate the median of the ratios. *C*, Time differences between the first and last sampling are shown; the bar represents the median.

For the investigation of time courses of intrathecal complement factors during BoDV-1 infections, follow-up CSF samples with sampling differences of >1 day were available for 7 patients diagnosed with BoDV-1 encephalitis (Figure 5*A*, Supplementary Figure 3). Ratios of CSF complement factor levels in the last (sampling 12–45 days after hospitalization) and first available sample (sampling 0–35 days after hospitalization) were computed for each of those 7 patients (Figure 5*B*). Median time difference between CSF sampling was 24 days (range: 6–39 days; Figure 5*C*). In >50% of these patients, levels of C1q, CFB, CFD, C3b_iC3b, C5, CFH, and CFI were increasing from the first to the last CSF sample as indicated by a median ratio >1 (Figure 5*B*). In all 7 patients, levels of C1q, MBL, C4b, C2, and CFH in the last available CSF sample were above the upper 95% confidence limit of the median of control samples. These data demonstrate a long-term elevation of complement factors in the CSF of patients with BoDV-1 encephalitis during the course of the disease.

### Complement C3 Is Deposited in Postmortem Brain Sections of Patients With BoDV-1 Encephalitis

**Figure 6.**
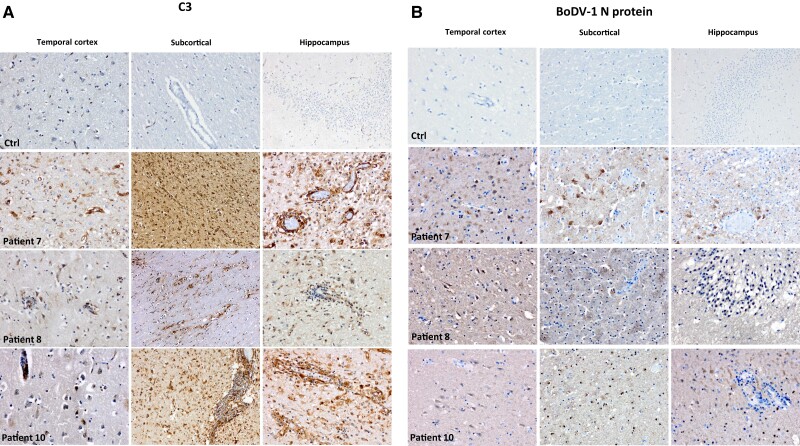
Immunohistochemistry reveals C3 complement deposits in brain tissue of patients with Borna disease virus 1 (BoDV-1) infection. Formalin-fixed, paraffin-embedded tissue slices of 3 patients with confirmed BoDV-1 infection and 1 patient without central nervous infection were stained by immunohistochemistry. *A*, Consistent with the extent of inflammation, deposition of C3 complement was scattered throughout the gray and subcortical white matter but was accentuated in perivascular spaces. Apart from the basal ganglia and the brain stem, the brain regions most affected by inflammation included the temporal cortex and the hippocampus. C3 complement was detected in some neurons of the neo- and allocortex, but was pronounced in the Virchow-Robin spaces and in inflammatory areas, where it was located in reactive astrocytes, activated microglia cells, and macrophages. The inflammation was accompanied by microglia nodules and a glial-mesenchymal reaction of the surrounding brain parenchyma. *B*, Immunohistochemistry with an antibody against the BoDV-1 N protein (Bo-18) revealed expression of the viral nucleoprotein in cell nuclei and cytoplasm. While in neocortical and hippocampal regions neurons were affected to varying degrees, immunoreactivity of glial cells and particularly reactive astrocytes (patient 7) was evident in the subcortical white matter.

**Figure 7.**
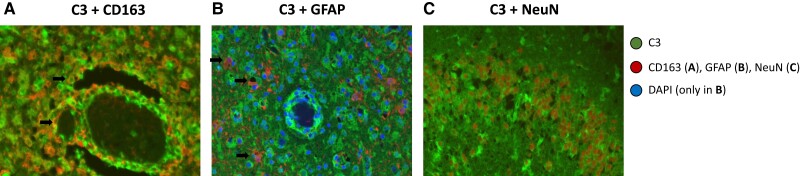
Double immunofluorescence demonstrates expression of C3 in microglial cells, astrocytes, and neurons. Formalin-fixed, paraffin-embedded brain tissue of patient 8 underwent double immunofluorescence staining. Opal 520 (green fluorescence) was used as a fluorochrome dye for the antibody directed against C3, while Opal 650 (red fluorescence) was applied for antibodies against CD163 (marker of microglial cells), glial fibrillary acidic protein (GFAP; marker of astrocytes), and neuronal nuclei (NeuN; marker of postmitotic neuronal cells), respectively. Examples of co-localizations are indicated by an arrow. *A*, CD163-highlighted activated, C3-immunoreactive microglial cells preferentially arranged in perivascular regions. *B*, Expression of C3 was also found in reactive astrocytes that were immunoreactive for GFAP. Cell nuclei were stained with DAPI (blue fluorescence). *C*, In the dentate gyrus, C3 was detected in NeuN-immunoreactive granular neuronal cells.

Formalin-fixed, paraffin-embedded brain tissue slices of 3 patients with confirmed BoDV-1 meningoencephalitis were stained by antibodies against human complement factor C3, which was preferentially deposited within vessel walls, in perivascular regions, activated microglia cells/macrophages, and reactive astrocytes (consistent with its proposed role as a marker for potentially proinflammatory neurotoxic reactive A1 astrocytes [20]) as well as in neurons (Figure 6*A*). Correspondingly, C3 co-localized with the microglial marker CD163, the glial antigen GFAP (as a marker of astrocytes), and the neuronal marker NeuN (Figure 7). To a lesser extent, C3 was randomly distributed in the interstitium of gray and subcortical white matter including basal ganglia. Virus-infected neurons, stained with an antibody directed against the BoDV-1 nucleoprotein, were found scattered throughout the brain (Figure 6*B*).

## DISCUSSION

Evidence of a role of the complement system in various neurological diseases is increasing [25]. With respect to viral infections of the CNS, an elevation and activation of certain complement factors have been shown for herpes simplex encephalitis and TBE: In herpes simplex encephalitis, increased intrathecal levels of C3a, C3b, C5, and C5a have been described [26]. C3a and C5a remained increased even in later stages and are probably linked to a prolonged inflammation. Another study examining patients with TBE found significantly elevated CSF levels of C1q, C3a, C3b, and C5a in comparison to controls [27]. For West Nile virus infection, a protective role of the complement system was described in a mouse model [28]. It was demonstrated that individual pathways of complement activation control West Nile virus infection by priming adaptive immune responses through distinct mechanisms. For flaviviruses in general, both protective and pathogenic roles of the complement system have been described depending on the specific virus, the phase of the infection, and the immune status of the host [29].

Studies addressing the complement system in human BoDV-1 infections have been lacking so far. The current knowledge about complement factors in animal models of BoDV-1 infection is also very limited. One study indirectly investigated the role of the complement system in BoDV-1–infected horses and rabbits by measuring complement-fixing antibodies, which were found in more than two-thirds of rabbit sera, but very rarely in horses [30]. Another study reported an enhanced expression of the complement factor C1q in the brains of rats experimentally infected with BoDV-1 [31]. In this study, cells expressing C1q were identified as microglial cells. As the expression of C1q was timely correlated with the development of neurological disease, the authors assumed a potential role in the pathogenesis of BoDV-1 encephalitis.

In the present study, we show an elevation of factors of all complement pathways as well as an activation of the complement cascade in CSF samples of patients with BoDV-1 infection. Astrocytes and microglia have been identified as sources of complement factors in various neurological diseases [21]. As potential sources of the elevated complement factors in CSF that we found in the present study, either an intrathecal synthesis or a passive diffusion over a leaky brain-blood barrier with synthesis in the liver can be considered, while co-localization of C3 deposits with GFAP in brain sections is more indicative for a synthesis within the CNS.

One aim of the present study was the evaluation of complement factors as possible biomarkers for BoDV-1 infections. Complement factors C4b and C2 were above the maximum of noninflammatory controls in all first available CSF samples of patients with BoDV-1 encephalitis, making these markers promising in order to differentiate BoDV-1 infections from noninflammatory conditions. In comparison to TBE, we found no significant differences with respect to the acute phase of the disease. Noticeable, however, was a long-term activation of the complement system in BoDV-1 patients. Between the first and the last available sample, we even observed an increase in C1q in 6 of 7 patients with BoDV-1 infection, whereby the last sample was collected 24 days (median; range: 6–39 days) after the first available sample. For our TBE cohort, only a single point measurement in the acute phase was available, but literature demonstrates a decline of C1q already 1 week after fever onset in TBE [27]. The same study found no influence of the enhanced complement cascade on the severity of acute disease or neurological sequelae in patients with TBE [27]. For patients with BoDV-1 encephalitis, we show a negative correlation of the concentration of certain complement factors in early available CSF samples with survival. This result supports the possible interpretation that high concentrations of complement factors could be harmful in human BoDV-1 infections. However, the intensive care unit setting and the individual decision of termination of life-prolonging intensive care treatment should be taken into consideration as a potential biasing factor for the reliability of correlations with survival in patients with BoDV-1 infection. Additionally, a trend toward a longer survival of patients with BoDV-1 under long-term immunosuppressive medication was observed in our data (Supplementary Figure 3*C*) and has been previously described [6]. However, neither short-term (steroids, intravenous immunoglobulins) nor long-term (anti-thymocyte globulin, azathioprine, basiliximab, cyclosporine A, everolimus, mycophenolate mofetil) immunosuppressive medication significantly influenced levels of complement factors in CSF of patients with BoDV-1 encephalitis, with the exception of factor C3 (Supplementary Figure 4*A* and 4*B*). Thus, use of immunosuppressive medication in patients with BoDV-1 encephalitis does not seem to explain interindividual differences in CSF complement levels.

So far, no approved therapy for BoDV-1 infections is available. In addition to immunosuppressive medication, favipiravir and ribavirin were administered to 3 of the patients whose CSF samples were included in this study, and 1 patient received favipiravir alone. For each of these compounds, an in vitro effect against BoDV-1 in cell culture has previously been demonstrated [32, 33], but unfortunately only 1 of the patients with BoDV-1 infection included in this study who receives antiviral and immunosuppressive medication has survived the infection. For specifically targeting the complement system, a number of preclinically and clinically used compounds are available [22]. As targeted anti-complement system therapy for paroxysmal nocturnal hemoglobinuria, myasthenia gravis, or NMOSD, inhibitors of complement factor C5 (eculizumab, ravulizumab) are in clinical use. Other substances are being evaluated in preclinical or clinical trials such as ANX005 (anti-C1q), sutimlimab (anti-C1s), AMY-101, and APL-9, which are cyclic peptides of the compstatin family (anti-C3), as well as vilobelimab (anti-C5a) and avdoralimab (anti-C5a receptor) [22, 34, 35]. As a therapeutic agent with a long history of clinical application, high-dose intravenous immunoglobulin is mainly thought to mediate its clinical effects through its interaction with C3 [22].

Given the possibility of a targeted complement inhibition and taking our negative correlation with survival into consideration, further investigations addressing the contribution of the complement system to severe BoDV-1 encephalitis with regard to tissue destruction and possible inhibition of viral spread are warranted. An animal model could help to address the point of whether an inhibition of the persistently activated complement cascade is harmful or beneficial for the outcome in BoDV-1 infections, as the complement system might be a double-edged sword.

## Supplementary Data

Supplementary materials are available at *The Journal of Infectious Diseases* online (http://jid.oxfordjournals.org/). Supplementary materials consist of data provided by the author that are published to benefit the reader. The posted materials are not copyedited. The contents of all supplementary data are the sole responsibility of the authors. Questions or messages regarding errors should be addressed to the author.

## Supplementary Material

jiae183_Supplementary_Data

## Notes

***Author contributions.*** M. B., B. S., S. B., and K. A. designed the study. K. A., B. N., D. L., E. E., D. T., and H. H. N. collected and characterized the samples. M. T. and L. E. performed the multiplex experiments and prepared the data. S. Z. performed and interpreted the immunohistochemistry. M. B. and S. Z. analyzed the data and created the figures. M. B. drafted and wrote the manuscript (first draft). B. S., S. Z., S. B., and K. A. reviewed and edited the manuscript. B. S., A. G., M. J. R., S. B., and K. A. supervised the study. All authors were involved in critically reading of the manuscript and approved the final version of the manuscript.

***Financial support*.** This study was kindly supported by the intramural research ReForM-A program of the Faculty for Medicine of the University of Regensburg/University Hospital Regensburg (to M. B.). The authors also thank the German Federal Ministry of Education and Research for funding the project “Bornavirus—Focal Point Bavaria” (grant number 01KI2002, to B. S.) as well as the Bavarian State Ministry of Health, Care, and Prevention for funding the project “Zoonotic Bornavirus Focal Point Bavaria (ZooBoFo)” (to B. S.). Funding to pay the Open Access publication charges for this article was provided by the Institute of Clinical Microbiology and Hygiene, University Hospital Regensburg, Regensburg, Germany.

## References

[1] Rubbenstroth D , Briese T, Dürrwald R, et al ICTV virus taxonomy profile: Bornaviridae. J Gen Virol 2021; 102:1613. 10.1099/jgv.0.001613 PMC8491894 34227935

[2] Hilbe M , Herrsche R, Kolodziejek J, Nowotny N, Zlinszky K, Ehrensperger F. Shrews as reservoir hosts of Borna disease virus. Emerg Infect Dis 2006; 12:675–7. 16704819 10.3201/eid1204.051418 PMC3294707

[3] Korn K , Coras R, Bobinger T, et al Fatal encephalitis associated with Borna disease virus 1. N Engl J Med 2018; 379:1375–7. 30281979 10.1056/NEJMc1800724

[4] Schlottau K , Forth L, Angstwurm K, et al Fatal encephalitic Borna disease virus 1 in solid-organ transplant recipients. N Engl J Med 2018; 379:1377–9. 30281984 10.1056/NEJMc1803115

[5] Coras R , Korn K, Kuerten S, Huttner HB, Ensser A. Severe Bornavirus-encephalitis presenting as Guillain-Barré-syndrome. Acta Neuropathol 2019; 137:1017–9. 30953131 10.1007/s00401-019-02005-z

[6] Niller HH , Angstwurm K, Rubbenstroth D, et al Zoonotic spillover infections with Borna disease virus 1 leading to fatal human encephalitis, 1999–2019: an epidemiological investigation. Lancet Infect Dis 2020; 20:467–77. 31924550 10.1016/S1473-3099(19)30546-8

[7] Eisermann P , Rubbenstroth D, Cadar D, et al Active case finding of current Bornavirus infections in human encephalitis cases of unknown etiology, Germany, 2018–2020. Emerg Infect Dis 2021; 27:1371–9. 33900167 10.3201/eid2705.204490 PMC8084505

[8] Tappe D , Pörtner K, Frank C, et al Investigation of fatal human Borna disease virus 1 encephalitis outside the previously known area for human cases, Brandenburg, Germany—a case report. BMC Infect Dis 2021; 21:787. 34376142 10.1186/s12879-021-06439-3 PMC8353434

[9] Neumann B , Angstwurm K, Linker RA, et al Antibodies against viral nucleo-, phospho-, and X protein contribute to serological diagnosis of fatal Borna disease virus 1 infections. Cell Rep Med 2022; 3:100499. 35106511 10.1016/j.xcrm.2021.100499 PMC8784767

[10] Grosse L , Lieftüchter V, Vollmuth Y, et al First detected geographical cluster of BoDV-1 encephalitis from same small village in two children: therapeutic considerations and epidemiological implications. Infection 2023; 51:1383–98. 36821024 10.1007/s15010-023-01998-w PMC9947883

[11] Nobach D , Bourg M, Herzog S, et al Shedding of infectious Borna disease virus-1 in living bicolored white-toothed shrews. PLoS One 2015; 10:e0137018. 26313904 10.1371/journal.pone.0137018 PMC4552160

[12] Hallensleben W , Schwemmle M, Hausmann J, et al Borna disease virus–induced neurological disorder in mice: infection of neonates results in immunopathology. J Virol 1998; 72:4379–86. 9557728 10.1128/jvi.72.5.4379-4386.1998 PMC109668

[13] Narayan O , Herzog S, Frese K, Scheefers H, Rott R. Pathogenesis of Borna disease in rats: immune-mediated viral ophthalmoencephalopathy causing blindness and behavioral abnormalities. J Infect Dis 1983; 148:305–15. 6604114 10.1093/infdis/148.2.305

[14] Stitz L , Soeder D, Deschl U, Frese K, Rott R. Inhibition of immune-mediated meningoencephalitis in persistently Borna disease virus–infected rats by cyclosporine A. J Immunol 1989; 143:4250–6. 2592774

[15] Gierend M , Ludwig H. Influence of immunosuppressive treatment of Borna disease in rabbits. Arch Virol 1981; 67:217–28. 7224860 10.1007/BF01318132

[16] Liesche F , Ruf V, Zoubaa S, et al The neuropathology of fatal encephalomyelitis in human Borna virus infection. Acta Neuropathol 2019; 138:653–65. 31346692 10.1007/s00401-019-02047-3 PMC6778062

[17] Rauch J , Steffen JF, Muntau B, et al Human Borna disease virus 1 encephalitis shows marked pro-inflammatory biomarker and tissue immunoactivation during the course of disease. Emerg Microbes Infect 2022; 11:1843–56. 35788177 10.1080/22221751.2022.2098831 PMC9336484

[18] Walport MJ . Complement. First of two parts. N Engl J Med 2001; 344:1058–66. 11287977 10.1056/NEJM200104053441406

[19] Stevens B , Allen NJ, Vazquez LE, et al The classical complement cascade mediates CNS synapse elimination. Cell 2007; 131:1164–78. 18083105 10.1016/j.cell.2007.10.036

[20] Liddelow SA , Guttenplan KA, Clarke LE, et al Neurotoxic reactive astrocytes are induced by activated microglia. Nature 2017; 541:481–7. 28099414 10.1038/nature21029 PMC5404890

[21] Veerhuis R , Nielsen HM, Tenner AJ. Complement in the brain. Mol Immunol 2011; 48:1592–603. 21546088 10.1016/j.molimm.2011.04.003 PMC3142281

[22] Dalakas MC , Alexopoulos H, Spaeth PJ. Complement in neurological disorders and emerging complement-targeted therapeutics. Nat Rev Neurol 2020; 16:601–17. 33005040 10.1038/s41582-020-0400-0 PMC7528717

[23] Haas B , Becht H, Rott R. Purification and properties of an intranuclear virus-specific antigen from tissue infected with Borna disease virus. J Gen Virol 1986; 67:235–41. 3080548 10.1099/0022-1317-67-2-235

[24] Herden C , Schluesener HJ, Richt JA. Expression of allograft inflammatory factor-1 and haeme oxygenase-1 in brains of rats infected with the neurotropic Borna disease virus. Neuropathol Appl Neurobiol 2005; 31:512–21. 16150122 10.1111/j.1365-2990.2005.00668.x

[25] Carpanini SM , Torvell M, Morgan BP. Therapeutic inhibition of the complement system in diseases of the central nervous system. Front Immunol 2019; 10:362. 30886620 10.3389/fimmu.2019.00362 PMC6409326

[26] Eriksson CE , Studahl M, Bergström T. Acute and prolonged complement activation in the central nervous system during herpes simplex encephalitis. J Neuroimmunol 2016; 295–96:130–8. 10.1016/j.jneuroim.2016.04.013 27235358

[27] Veje M , Studahl M, Bergström T. Intrathecal complement activation by the classical pathway in tick-borne encephalitis. J Neurovirol 2019; 25:397–404. 30850976 10.1007/s13365-019-00734-1 PMC6647885

[28] Mehlhop E , Diamond MS. Protective immune responses against West Nile virus are primed by distinct complement activation pathways. J Exp Med 2006; 203:1371–81. 16651386 10.1084/jem.20052388 PMC2121216

[29] Avirutnan P , Mehlhop E, Diamond MS. Complement and its role in protection and pathogenesis of flavivirus infections. Vaccine 2008; 26(Suppl 8):I100–7. 19388173 10.1016/j.vaccine.2008.11.061 PMC2768071

[30] Hv S . Untersuchungen über den Nachweis von komplementbindenden Antikörpern bei Bornavirus-infizierten Pferden und Kaninchen. Zentralbl Veterinaermed 1954; 1:870–7.

[31] Dietzschold B , Schwaeble W, Schäfer MK, et al Expression of C1q, a subcomponent of the rat complement system, is dramatically enhanced in brains of rats with either Borna disease or experimental allergic encephalomyelitis. J Neurol Sci 1995; 130:11–6. 7544401 10.1016/0022-510x(94)00269-t

[32] Tokunaga T , Yamamoto Y, Sakai M, Tomonaga K, Honda T. Antiviral activity of favipiravir (T-705) against mammalian and avian bornaviruses. Antiviral Res 2017; 143:237–45. 28465146 10.1016/j.antiviral.2017.04.018

[33] Reuter A , Horie M, Höper D, et al Synergistic antiviral activity of ribavirin and interferon-α against parrot bornaviruses in avian cells. J Gen Virol 2016; 97:2096–103. 27439314 10.1099/jgv.0.000555

[34] Ram Kumar Pandian S , Arunachalam S, Deepak V, Kunjiappan S, Sundar K. Targeting complement cascade: an alternative strategy for COVID-19. 3 Biotech 2020; 10:479. 10.1007/s13205-020-02464-2 PMC7571295 33088671

[35] Bauer M , Weyland A, Marx G, et al Efficacy and safety of vilobelimab (IFX-1), a novel monoclonal anti-C5a antibody, in patients with early severe sepsis or septic shock—a randomized, placebo-controlled, double-blind, multicenter, phase IIa trial (SCIENS Study). Crit Care Explor 2021; 3:e0577. 34806021 10.1097/CCE.0000000000000577 PMC8601347

