# Supplementary material for: Long-term Elevation of Complement Factors in Cerebrospinal Fluid of Patients With Borna Disease Virus 1 Encephalitis
Source: J Infect Dis. 2024 Apr 9;230(4):e943–53. doi: 10.1093/infdis/jiae183 (PMC11481329; doi:10.1093/infdis/jiae183)
Supplement: jiae183_Supplementary_Data [file jiae183_supplementary_data.zip › Supplemental Figure S3_Bauswein et al..pptx]

## Slide 1
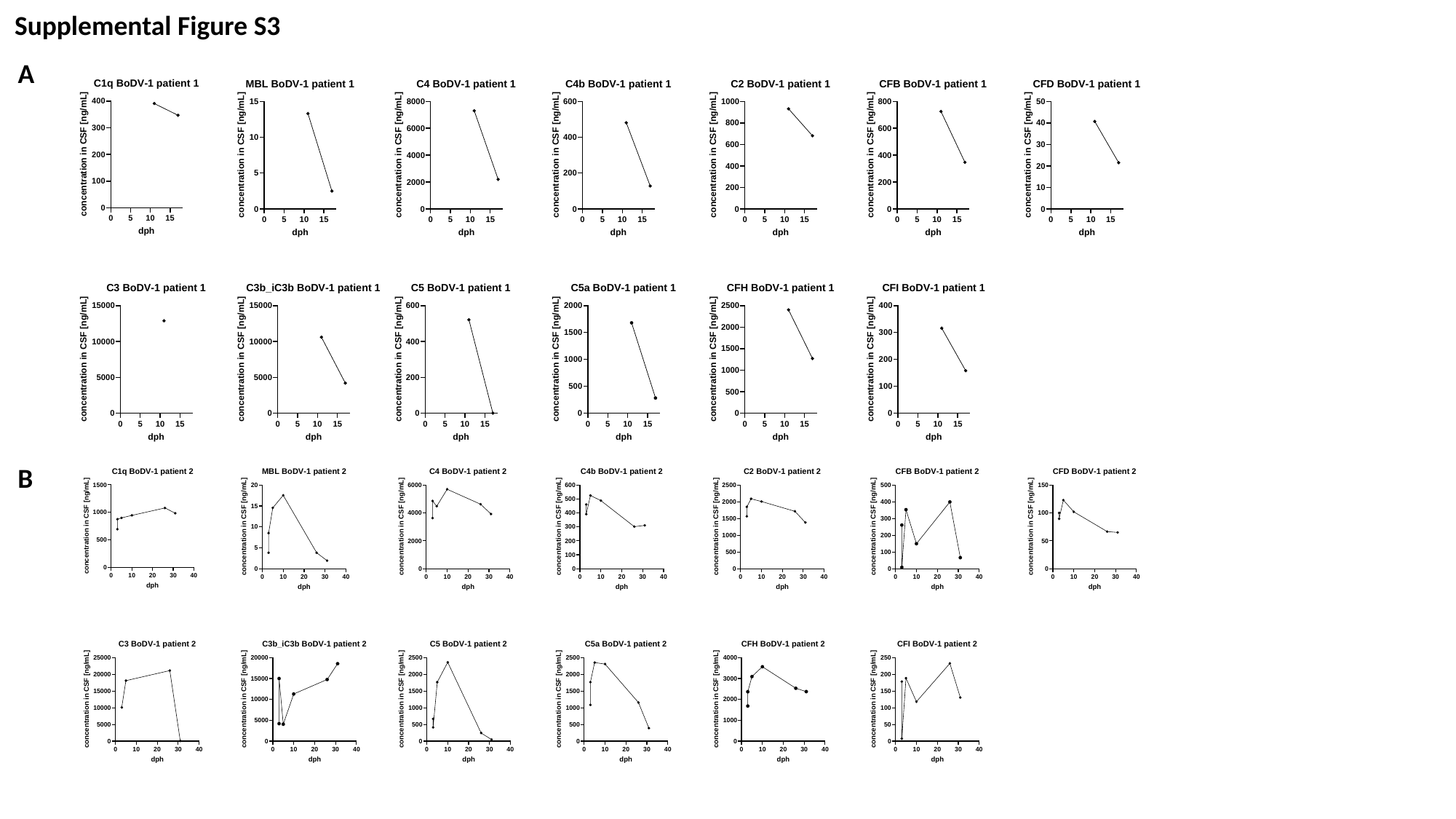

Supplemental Figure S3
A
B

## Slide 2
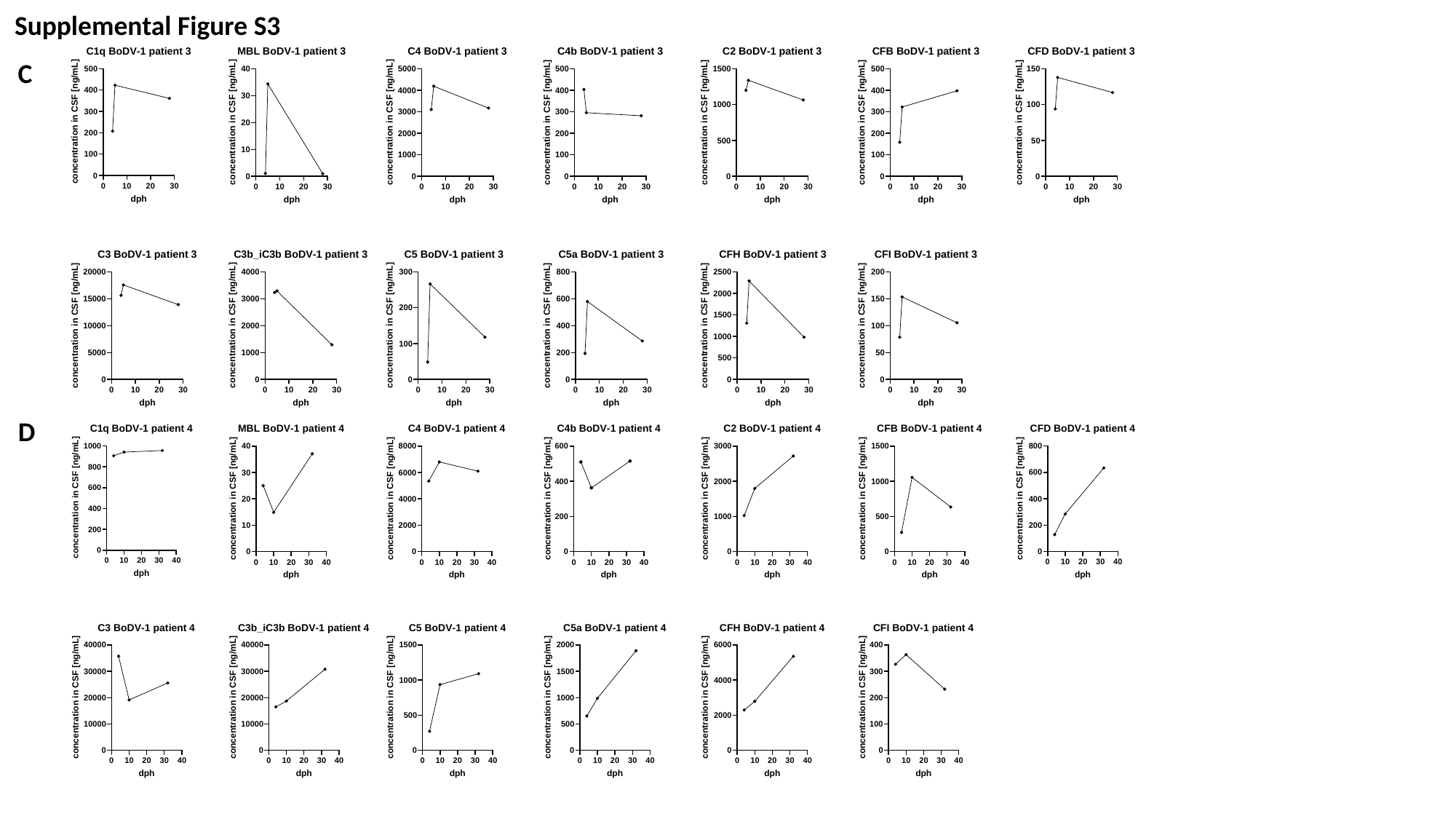

Supplemental Figure S3
C
D

## Slide 3
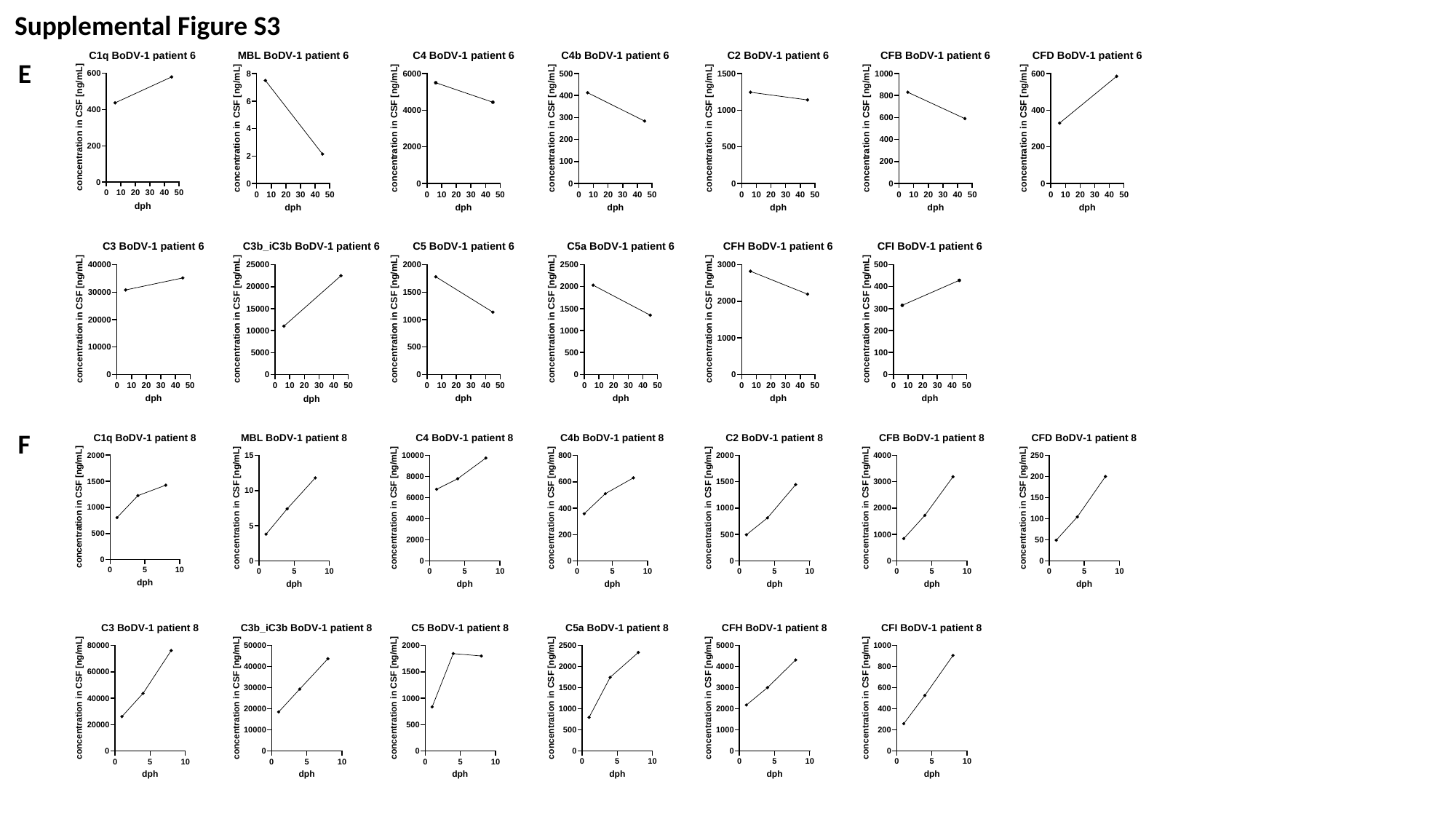

Supplemental Figure S3
E
F

## Slide 4
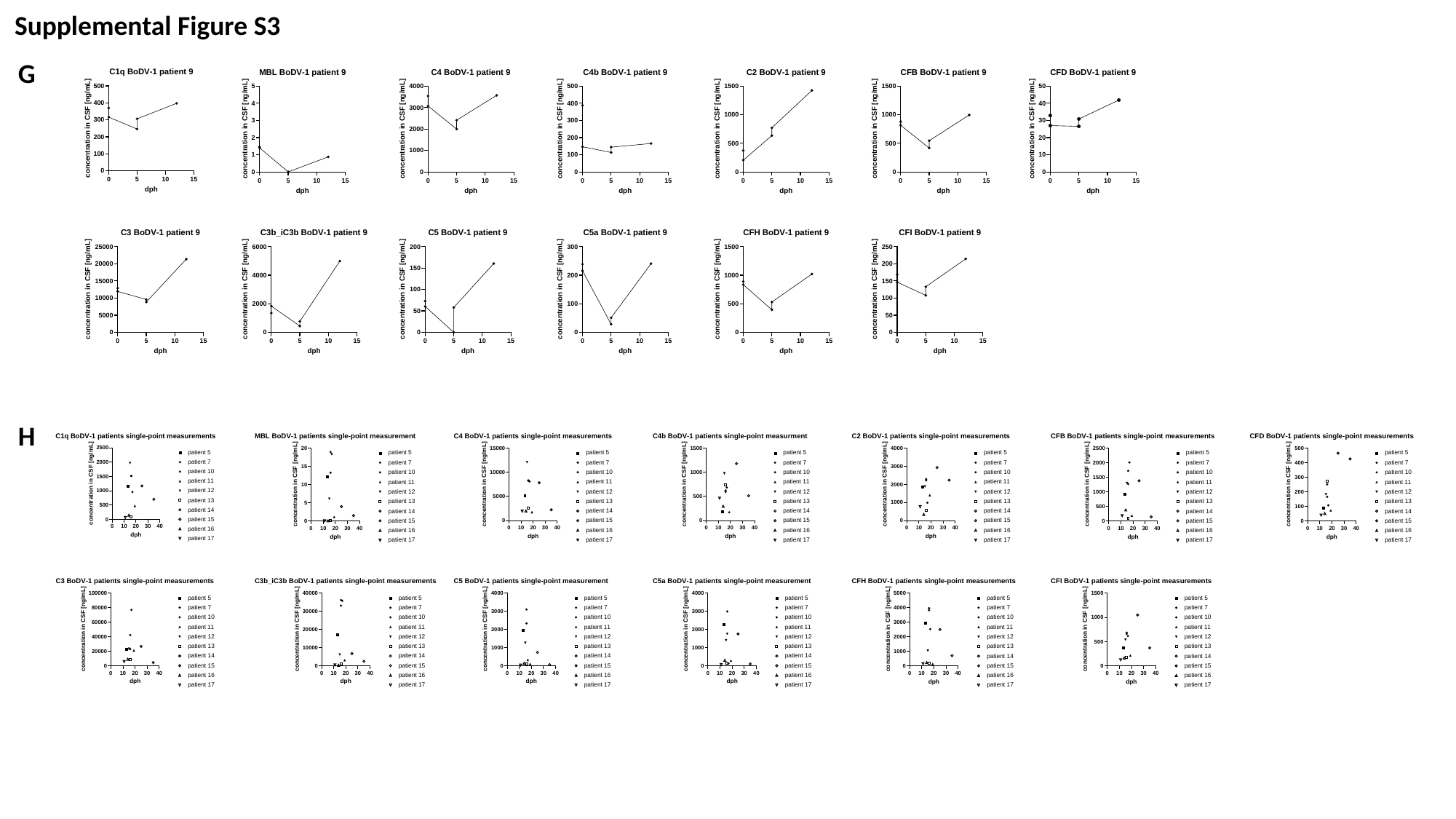

Supplemental Figure S3
G
H
