# Supplementary material for: Long-term Elevation of Complement Factors in Cerebrospinal Fluid of Patients With Borna Disease Virus 1 Encephalitis
Source: J Infect Dis. 2024 Apr 9;230(4):e943–53. doi: 10.1093/infdis/jiae183 (PMC11481329; doi:10.1093/infdis/jiae183)
Supplement: jiae183_Supplementary_Data [file jiae183_supplementary_data.zip › Supplemental Material_Bauswein et al._clean version_26.03..docx]

**Supplemental Material**

**Supplemental Table S1: Characteristics of patients with Borna disease virus 1 (BoDV-1) encephalitis**

| ID | REF literature | Age group [years] | Underlying disease | Short-term immunosuppression/immunomodulation | Long-term immunosuppression | Off-label antiviral treatment attempt | Survival  [days post hospitalization] |
| --- | --- | --- | --- | --- | --- | --- | --- |
| 1 | Niller 2020 #1 | 40−50 |  |  |  |  | 46 |
| 2 | Niller 2020 #2 | 30−40 |  |  |  |  | 57 |
| 3 | Niller 2020 #3 | 60−70 | solid-organ transplantation: heart |  | tacrolimus, azathioprin |  | 67 |
| 4 | Niller 2020 #4 | 40−50 | solid-organ transplantation: kidney |  | cyclosporine A, mycophenolate mofetil,  everolimus, tacrolimus, steroids |  | 53 |
| 5 | Niller 2020 #5 | 30−40 |  | intravenous immunoglobulin, steroids, immune adsorption, cyclophosphamide |  |  | 27 |
| 6 | Schlottau 2018 #1 | 60−70 | solid-organ transplantation: kidney | intravenous immunoglobulin | basiliximab, tacrolimus, mycophenolate mofetil, steroids, anti-thymocyte globulin, everolimus |  | 78 |
| 7 | Niller 2020 #8 | 50−60 |  | steroids, immune adsorption |  |  | 16 |
| 8 | Neumann 2022 | 70−80 |  |  |  |  | 15 |
| 9 | Eidenschink 2023 | 10−20 |  | intravenous immunoglobulin, steroids, cyclosporine A |  | ribavirin, favipiravir | 123 |
| 10 | Bauswein 2023 | 50−60 |  | intravenous immunoglobulin, steroids |  |  | 29 |
| 11 | Eisermann 2021 #3 | 10−20 |  | yes |  | ribavirin, favipiravir | 22 |
| 12 | unpublished | 60−70 |  | yes |  |  | 33 |
| 13 | Frank 2022 #1 | 50−60 |  |  | yes | favipiravir | 500 |
| 14 | Frank 2022 #2 | 70−80 |  | yes |  |  | 35 |
| 15 | Eisermann 2021 #4 | 70−80 |  | yes |  |  | 30 |
| 16 | unpublished | 60−70 |  | yes |  |  | 42 |
| 17 | Meyer 2022 | 10−20 |  |  | yes | ribavirin, favipiravir | > 550 |

**Supplemental Methods**

***Patients and samples***

Thirty-five diagnostic left-over CSF samples of 17 individual patients with confirmed BoDV-1 infection were included in the analysis (Table S1). Twenty-eight samples of ten individual patients were available at the Institute of Clinical Microbiology and Hygiene of the University Hospital Regensburg, Germany, seven samples of seven additional patients were provided by the Bernhard Nocht Institute for Tropical Medicine, Hamburg, Germany. Samples were collected between 1999 and 2022. Patients 1 to 5 of this publication correspond to patients 1 to 5 published by Niller et al. [1]. Patient 6 is the kidney transplant recipient 1 published by Schlottau et al. [2]. Patient 7 corresponds to patient 8 published by Niller et al. [1]. Patient 8 is the patient published by Neumann et al. [3], patient 9 is the patient published by Eidenschink et al. [4], patient 10 is the patient published by Bauswein et al. [5]. Patient 11 is the patient 3 published by Eisermann et al. [6], patient 13 and patient 14 are the patients 1 and 2 published by Frank et al. [7], patient 15 is the patient 4 published by Eisermann et al. [6], patient 17 is the patient published by Meyer et al. [8]. Patients 12 and 16 are so far unpublished cases.

As controls, 11 diagnostic left-over CSF samples of 11 individual patients who underwent diagnostic lumbar puncture in the years from 2020 to 2021 for the exclusion of an infection of the central nervous system were used. All CSF samples had been sent for a diagnostic microbiological work-up to the Institute of Clinical Microbiology and Hygiene of the University Hospital Regensburg, Germany. Indications for the lumbar puncture were exclusion of neuroborreliosis, herpes simplex virus (HSV), varizella zoster virus (VZV), cytomegaly virus (CMV), Epstein-Barr virus (EBV) or enterovirus infection. All samples showed a normal cell count (< 5 cells/µL), a normal lactate level (< 2.45 mmol/L) as well as a normal protein concentration (< 450 mg/L) and were unremarkable in microbiological work-up as required by the neurologists. Known or strongly suspected autoimmune diseases of the peripheral or central nervous system such as multiple sclerosis or chronic inflammatory polyneuroradiculopathy were exclusion criteria for control patients. The control group consisted of six patients with psychiatric disorders (3x somatoform disorder, 1x psychosis, 1x depression, 1x mild cognitive impairment), four patients with non-inflammatory diseases (transient ischemic attack, spinal muscular atrophy, polyneuropathy, focal epilepsy) and one patient with isolated papillitis.

***Bead-based multiplex assay for complement factors***

Complement factors in CSF samples were measured using the MILLIPLEX® Human Complement Magnetic Bead Panel 1 (Merck, Darmstadt, Germany; #HCMP1MAG-19K-07: C2, C4b, C5, C5a, CFD, CFI, MBL), the MILLIPLEX® Human Complement Magnetic Bead Panel 2 (Merck; #HCMP2MAG-19K-06: C1q, C3, C3b_iC3b, C4, CFB, CFH) and the MILLIPLEX® Human Complement Magnetic Bead Panel 2 (Merck; #HCMP2MAG-19K-01: C3) according to the manufacturer´s instructions. In brief, wells of the 96-well plate were prepared by adding 200 µL of wash buffer into each well. After incubation on a plate shaker for 10 minutes at room temperature (RT), wash buffer was decanted and either 25 µL of assay buffer (background), 25 µL of prepared working standards (6x serial 1:3 dilution in assay buffer), 25 µL of controls (diluted in assay buffer) or 25 µL of diluted CSF samples (dilution in assay buffer) were added to the wells.

For CSF samples, the following dilutions were used:

• C2, C4b, C5, C5a, CFD, CFI, MBL: 1:10/1:20

• C1q, C4, CFB, CFH: 1:100/1:200

• C3b_i3Cb: 1:100/1:200; 1:2,500

• C3: 1:25; 1:100/1:200; 1:1,000; 1:2,500; 1:10,000

Subsequently, 25 µL of the mixed beads were added to each well. The plate was then incubated on a plate shaker overnight at 2−8° C. Well contents were removed and the plate was washed three times with 200 µL of wash buffer before 50 µL of detection antibodies were added in each well. After incubation on a plate shaker for 1 hour at RT, 50 µL of streptavidin-phycoerythrin were added to each well and the plate was incubated on a plate shaker for another 30 minutes at RT. Well contents were removed and each well was washed three times with 200 µL of wash buffer. Then 150 µL of drive fluid were added to each well and beads were resuspended on a plate shaker for 5 minutes. The measurement was run on a MAGPIX® instrument (Luminex, Austin, TX, USA). Data were collected and the median fluorescent intensity (MFI) was analyzed with the xPONENT® software (Luminex). A 4- or 5-parameter logistic regression standard curve was fitted for calculating analyte concentrations in samples. For C3b_iC3b, the concentration of a measurement with an MFI within the quasi-linear range of the standard curve was selected. For C3, the mean concentration was calculated and used for further analysis if measurements of more than one dilution were within the quasi-linear range of the standard curve.

***Statistical analysis***

Differences in component factors within the groups (Ctrl, TBE, BoDV-1) were analyzed by Kruskal-Wallis tests, followed by Dunn´s multiple comparisons test, using GraphPad Prism version 10.1.0 (GraphPad Software, San Diego, CA, USA). A principal component analysis (PCA) was computed with R version 4.3.1 (The R Foundation for Statistical Computing, Vienna, Austria) using the prcomp() function. Encompassing ellipses for the groups were computed using the stat_ellipse() function of the ggplot2 package in R, based at a confidence level of 0.6. For correlation analysis, Spearman correlation coefficients with 95% confidence intervals (CI) were computed using GraphPad Prism. In addition, linear regression (with 95% confidence band of the best-fit line) was performed.

The following levels of significance were used: * p ≤ 0.05; ** p ≤ 0.01; *** p ≤ 0.001; **** p ≤ 0.0001; not significant (ns) p > 0.05

**Supplemental Figures**

**Supplemental Figure S1:**

**Evaluation of potential biases for correlation analysis between concentration of complement factors and survival.**

To investigate for a potential sampling bias, a Spearman correlation analysis was performed for the survival post hospitalization (x-axis) and the sampling of the first available CSF sample (y-axis). No significant correlation was found, making a sampling bias for correlation analysis between concentration of complement factors and survival unlikely.

**Supplemental Figure S2:**

**Intrathecal complement levels in BoDV-1 encephalitis are negatively correlated with survival in the sub-group of patients of center 1.**

Survival after hospital admission is shown on the x-axis, while concentrations of complement factors in the first available CSF samples (range 0−17 days after hospitalization) of patients with BoDV-1 encephalitis of center 1 only are given on the y-axis. Spearman correlation tests and a linear regression (black line; dotted lines represent 95% confidence band of best-fit line) were performed.

**Supplemental Figure S3:**

**Time courses of complement factors in CSF of patients with BoDV-1 encephalitis.**

Concentrations of complement factors in CSF are given for seven patients with available consecutive CSF samples (patients 1−4, 6, 8−9) and for patients with single-point measurements (patients 5, 7, 10−17).

**Supplemental Figure S4:**

**Immunosuppression does not affect the concentrations of complement factors in CSF of patients with BoDV-1.**

**A+B**: Sub-group analyses of patients with confirmed BoDV-1 infection were performed based on the variable of short- or long-term immunosuppression. For three patients (11 samples), no immunosuppressive medication was reported. Nine patients (14 samples) received steroids and intravenous immunoglobulin as short-term immunosuppressive/immunomodulatory medication. Five patients (10 samples) received or had received long-term immunosuppression (e. g. anti-thymocyte globulin, azathioprine, basiliximab, cyclosporine A, everolimus, mycophenolate mofetil) due to another underlying disease (solid-organ transplantation). Medians with 95% confidence interval are shown for only the first available CSF samples (**A**) and all available samples (**B**), respectively. For statistical analysis, an ordinary two-way ANOVA was performed, followed by Tukey´s multiple comparisons test. The given asterisk indicates a significant difference, non-significant differences are not shown. **C**: While the survival post hospitalization of short-term immunosuppressed patients did not significantly differ from patients without record of immunosuppression, patients with long-term immunosuppressive medication tended to live longer (* p ≤ 0.05; ns p > 0.05).

**References**

1. Niller HH, Angstwurm K, Rubbenstroth D, et al. Zoonotic spillover infections with Borna disease virus 1 leading to fatal human encephalitis, 1999–2019: an epidemiological investigation. The Lancet Infectious Diseases **2020**; 20:467–77.

2. Schlottau K, Forth L, Angstwurm K, et al. Fatal Encephalitic Borna Disease Virus 1 in Solid-Organ Transplant Recipients. N Engl J Med **2018**; 379:1377–9.

3. Neumann B, Angstwurm K, Linker RA, et al. Antibodies against viral nucleo-, phospho-, and X protein contribute to serological diagnosis of fatal Borna disease virus 1 infections. Cell Rep Med **2022**; 3:100499.

4. Eidenschink L, Knoll G, Tappe D, et al. IFN-γ-Based ELISpot as a New Tool to Detect Human Infections with Borna Disease Virus 1 (BoDV-1): A Pilot Study. Viruses **2023**; 15:194.

5. Bauswein M, Eidenschink L, Knoll G, et al. Human Infections with Borna Disease Virus 1 (BoDV-1) Primarily Lead to Severe Encephalitis: Further Evidence from the Seroepidemiological BoSOT Study in an Endemic Region in Southern Germany. Viruses **2023**; 15:188.

6. Eisermann P, Rubbenstroth D, Cadar D, et al. Active Case Finding of Current Bornavirus Infections in Human Encephalitis Cases of Unknown Etiology, Germany, 2018-2020. Emerg Infect Dis **2021**; 27:1371–9.

7. Frank C, Wickel J, Brämer D, et al. Human Borna disease virus 1 (BoDV-1) encephalitis cases in the north and east of Germany. Emerg Microbes Infect **2022**; 11:6–13.

8. Meyer T, Tappe D, Hasan D, et al. „Borna disease virus 1“(BoDV-1)-Enzephalitis eines 18-Jährigen außerhalb des bisher bekannten Endemiegebietes. DGNeurologie **2022**; 5:300–4.
