# Supplementary figures and images for: Long-term Elevation of Complement Factors in Cerebrospinal Fluid of Patients With Borna Disease Virus 1 Encephalitis
Source: J Infect Dis. 2024 Apr 9;230(4):e943–53. doi: 10.1093/infdis/jiae183 (PMC11481329; doi:10.1093/infdis/jiae183)

## Slide 1
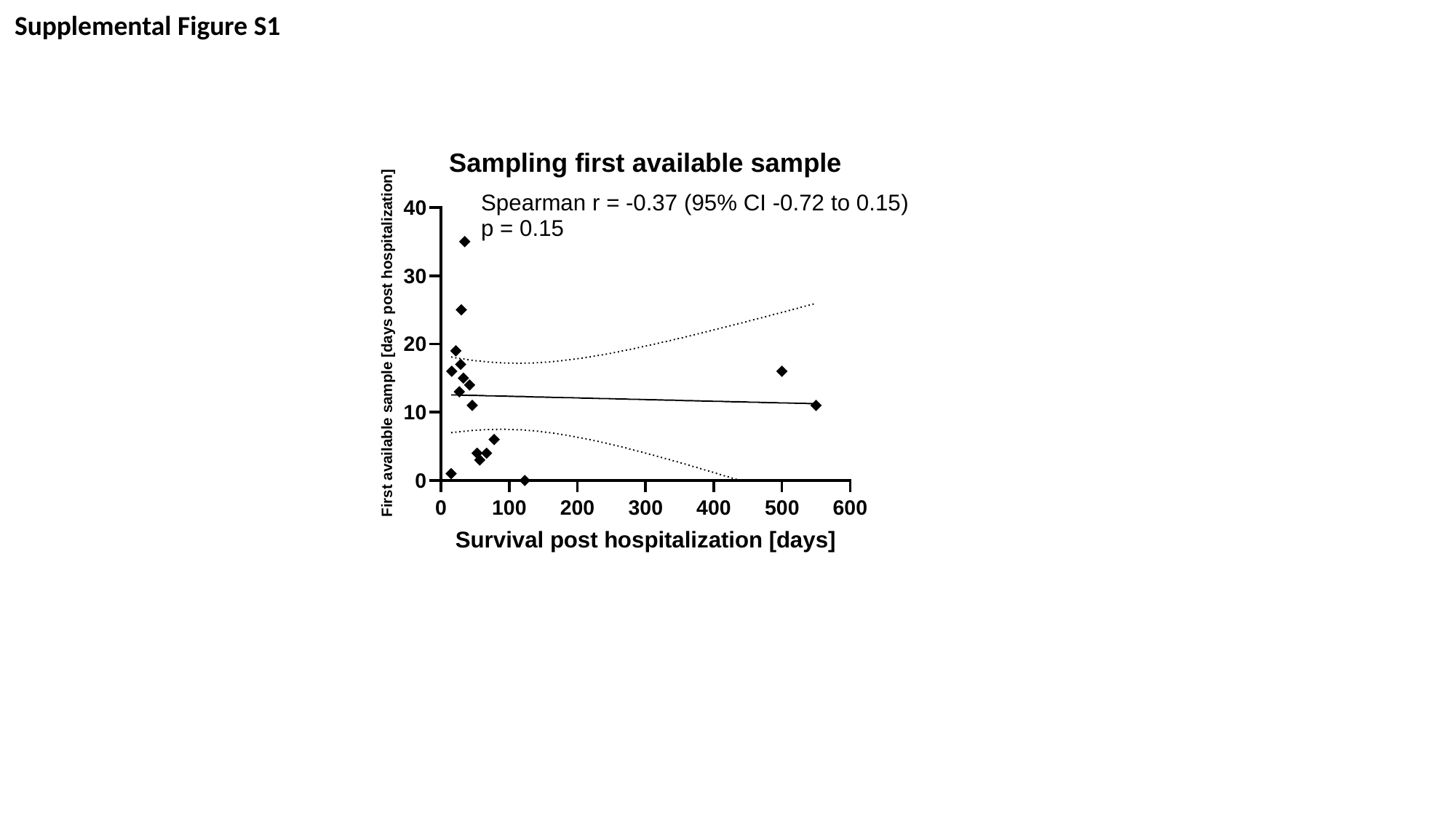

Supplemental Figure S1

Supplement: jiae183_Supplementary_Data [file jiae183_supplementary_data.zip › Supplemental Figure S1_Bauswein et al..pptx]

## Slide 1
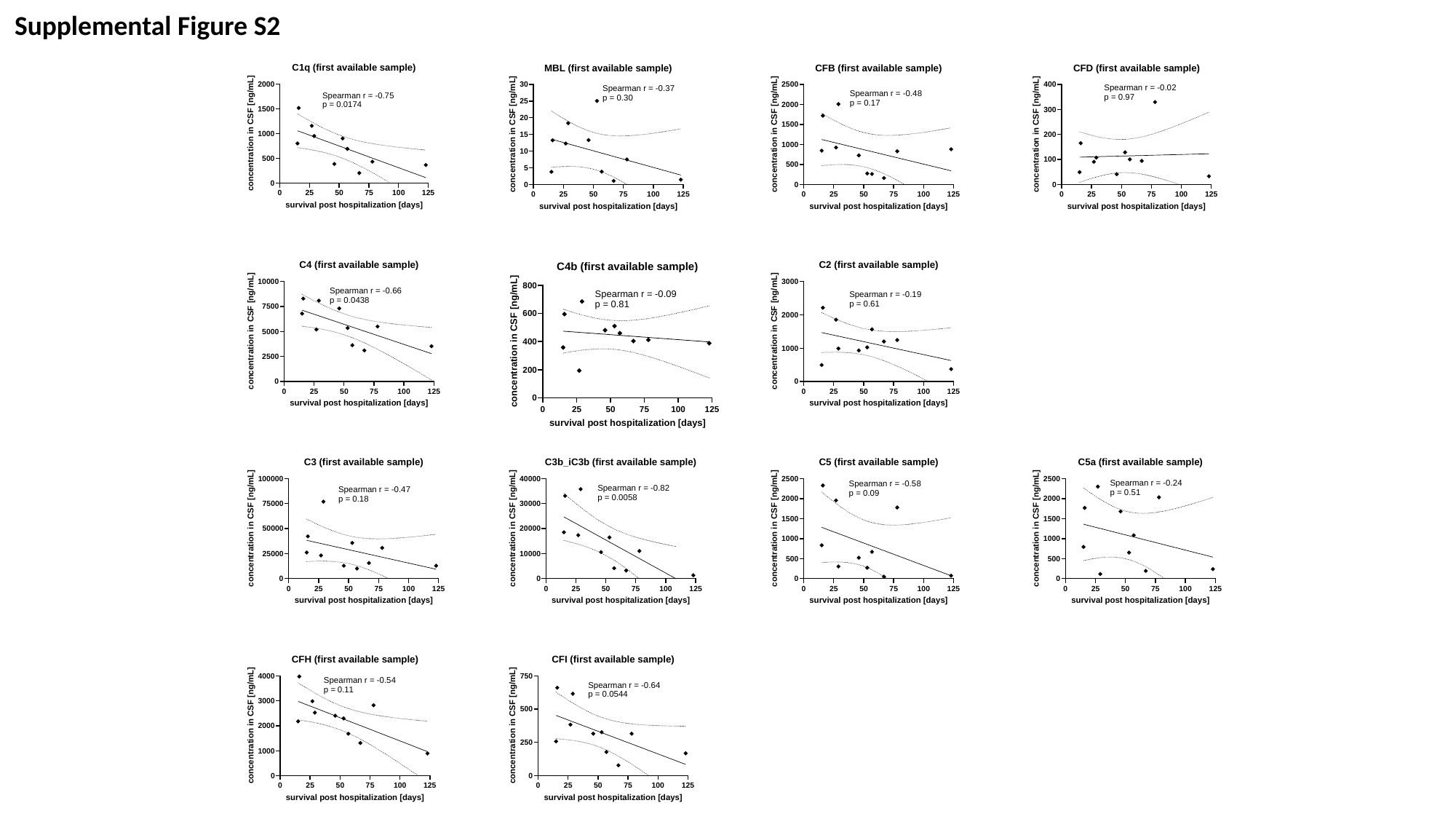

Supplemental Figure S2

Supplement: jiae183_Supplementary_Data [file jiae183_supplementary_data.zip › Supplemental Figure S2_Bauswein et al..pptx]

## Slide 1
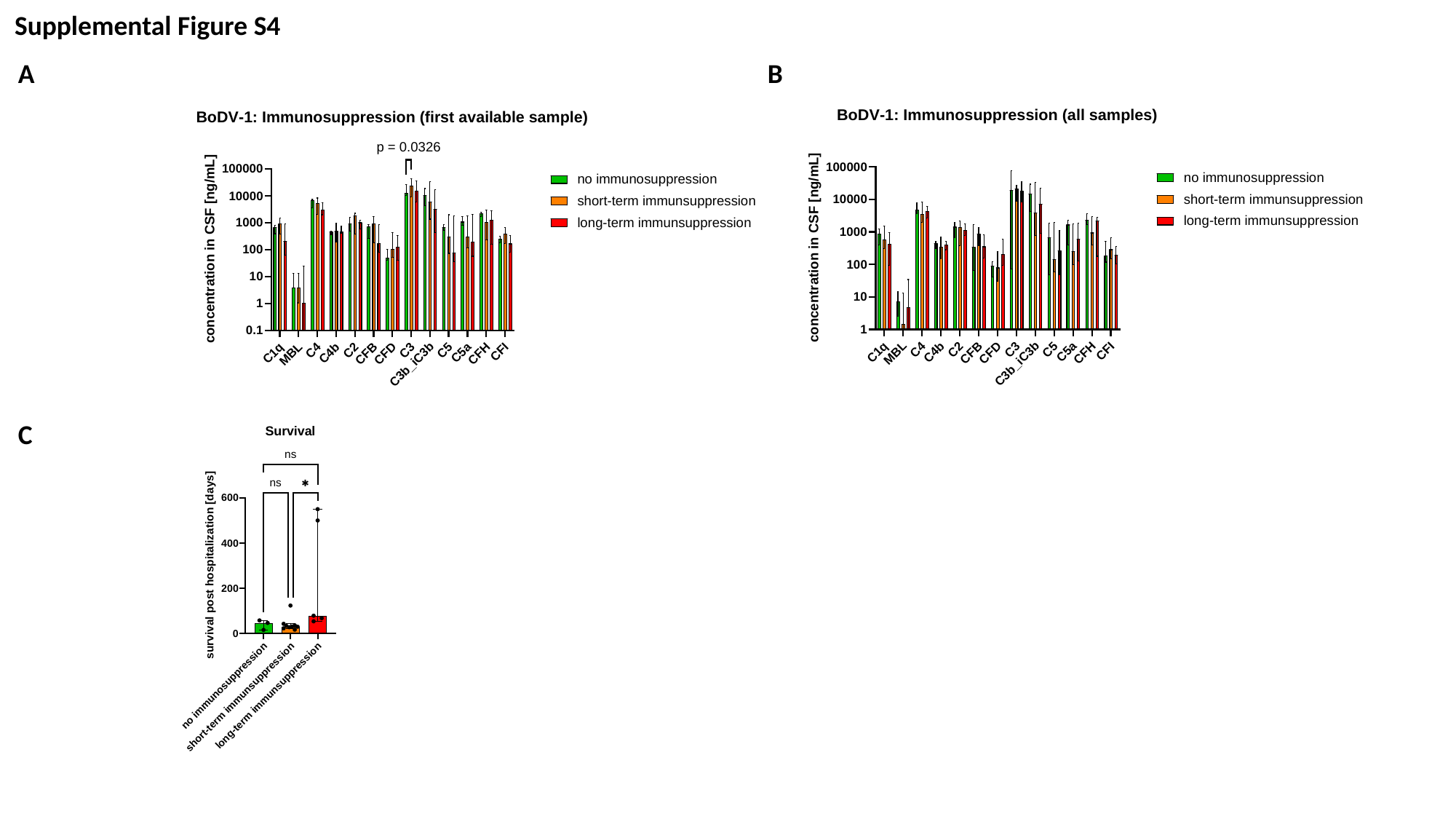

Supplemental Figure S4
A
B
C

Supplement: jiae183_Supplementary_Data [file jiae183_supplementary_data.zip › Supplemental Figure S4_Bauswein et al..pptx]
